# Internet-delivered therapist-assisted cognitive behavioral therapy for gambling disorder: a randomized controlled trial

**DOI:** 10.3389/fpsyt.2023.1243826

**Published:** 2023-12-11

**Authors:** Mikael Mide, Jessica Mattiasson, David Norlin, Helena Sehlin, Josefine Rasmusson, Sofia Ljung, Amanda Lindskog, Jonna Petersson, Fanny Saavedra, Anna Söderpalm Gordh

**Affiliations:** ^1^Department of Addiction Medicine, Institute of Neuroscience and Physiology, Section of Psychiatry and Neurochemistry, Sahlgrenska Academy, University of Gothenburg, Gothenburg, Sweden; ^2^Department of Addiction and Dependency, Sahlgrenska University Hospital, Gothenburg, Sweden

**Keywords:** online, CBT, ICBT, gambling problems, RCT

## Abstract

**Objectives:**

Cognitive behavioral therapy (CBT) is the most promising treatment for gambling disorder (GD) but only 21% of those with problematic gambling seek treatment. CBT over the Internet might be one way to reach a larger population. The aim of this study was to assess the effectiveness of Internet-delivered CBT with therapist guidance compared to an active control treatment.

**Methods:**

Using a single-blinded design, 71 treatment-seeking gamblers (18–75 years) diagnosed with GD were randomized to 8 weeks of Internet-delivered CBT guided by telephone support, or 8 weeks of Internet-delivered motivational enhancement paired with motivational interviewing via telephone (IMI). The primary outcome was gambling symptoms measured at a first face-to-face assessment, baseline (treatment start), every 2 weeks, post-treatment, and 6-month follow-up. Gambling expenditures, time spent gambling, depression, anxiety, cognitive distortions, and quality of life were assessed as secondary outcomes. Analysis was performed on the full analysis sample (*n* = 60), with intention-to-treat sensitivity analyses (*n* = 69).

**Results:**

In the CBT group, 80% stayed in treatment until the final week, compared to 67% in IMI. Post-treatment and at 6-month follow-up, no differences were found between CBT and IMI for any outcome measure. An exploratory analysis of the total sample (*n* = 60) showed a significant effect of time during treatment on gambling symptoms (d, [95% CI] 0.52, [−1.15, 2.02]) and all secondary outcomes except the gambling diary (depression (0.89, [−1.07–2.65]); anxiety (0.69, [−1.20–2.38])); cognitive distortions (0.84, [−0.73–2.29]); quality of life (0.60, [−0.61–1.70])). Post-treatment, there were no clinical gambling symptoms in either group. Some deterioration was seen between post-treatment and 6-month follow-up on gambling symptoms (0.42, [−1.74–2.43]), depression (0.59, [−0.82–1.86]), and anxiety (0.30, [−0.99–1.48]). Additionally, it was observed that the largest reduction in gambling symptoms was between the first assessment and baseline.

**Conclusion:**

Both treatments offered in this study were effective at reducing gambling symptoms. It is also possible that the process of change started before treatment, which gives promise to low-intensity interventions for GD. Additional research is needed as this approach could be both cost-effective and has the potential to reach more patients in need of treatment than is currently possible.

**Clinical trial registration:**

https://www.isrctn.com/, identifier ISRCTN38692394.

## Introduction

In Sweden, around 1.3% of the population between 16 and 87 years old have some degree of gambling problems, and 0.6% have severe problems (1). However, prevalence varies considerably between countries from 0.5% to 7.6% (2). Gambling problems that become severe enough are considered a gambling disorder (GD). This is defined in the Diagnostic and Statistical Manual Version 5 (DSM-5) as a persistent gambling behavior manifesting as at least four of the following nine criteria during the past year: (1) the need to gamble for increasing amounts of money, (2) restlessness or irritability when attempting to cut down on gambling, (3) repeated unsuccessful attempts to stop or control one’s gambling behavior, (4) preoccupation with gambling, (5) gambling when feeling distressed, (6) chasing losses (gambling more to “get even” after losing money), (7) lies to conceal the extent of gambling, (8) jeopardized or lost significant relationships, job, or educational and career opportunities, and (9) relies on others for financial bailouts (3). Depending on the number of criteria fulfilled, GD can be classified as a mild (4 - 5 criteria), moderate (6 - 7 criteria), and severe (8 - 9 criteria) disorder. Another definition can be found in the ICD-11 where a gambling disorder is defined as a persistent and recurring gambling behavior characterized by (1) impaired control over gambling, (2) gambling being prioritized over other life interests and daily activities, and (3) continuation or escalation of gambling despite negative consequences (4).

In addition to gambling symptoms, GD is also associated with a number of other problems and consequences, such as financial problems and difficulties in close relationships (5), heightened rates of suicide attempts and suicidal ideation (6), and higher mortality rates (7). Furthermore, meta-analytic approaches have also shown high prevalence rates of co-morbid psychiatric disorders in GD populations seeking treatment: mood disorders (23.1%), anxiety disorders (17.6%), nicotine dependence (56.4%), alcohol use disorder (21.1%), illicit drug use disorder (7.0%), attention-deficit hyperactivity disorder (9.3%), and any personality disorder (47.9%) (8, 9). Thus, there is clearly a need for effective treatments for GD. Thus far, there is no single gold-standard treatment for GD. The most promising and extensively studied treatment for GD to date is cognitive behavioral therapy (CBT). Face-to-face CBT has shown larger effects than other psychological treatments for GD on gambling symptoms, and the evidence supporting CBT is currently stronger than for other psychological treatments (10) and pharmacological treatments (11). However, although promising, the long-term effects of CBT are less known as there is a lack of follow-up studies (10, 12) and treatment effects might be overestimated due to publication bias (12). Overall, CBT is estimated to have a large effect on reducing gambling severity, moderate effects on gambling frequency and anxiety, and small effects on gambling intensity, depressive symptoms (10, 12), and quality of life (13). Effects were generally larger when the treatment was delivered face-to-face (10). Apart from CBT, the most studied psychological intervention for GD has been motivational interviewing (MI). A recent meta-analysis found some support for MI in combination with CBT but no significant effect of MI as a standalone treatment (10).

There are several different CBT-based treatment programs for GD, and the contents can vary somewhat between treatments. Commonly featured interventions are identifying and managing triggers to gamble, cognitive restructuring of gambling-related cognitive distortions, and focusing on alternative activities to gambling (14). CBT can also be delivered to patients in a number of ways, i.e., by meeting a therapist individually face-to-face, in a group format, or via the Internet. Internet-delivered CBT (ICBT) consisting of “modules” including text, questionnaires, and different exercises delivered to patients each week over the course of treatment has proved effective for a number of different psychiatric disorders such as depression (15–17), anxiety disorders (18, 19), and insomnia (20). In addition, it has also been found effective at reducing distress and functional impairment in various chronic somatic conditions (21), further establishing the relevance of the treatment format. In addition, in direct comparisons of ICBT with CBT delivered face-to-face, ICBT has generally performed equally well as its more traditional face-to-face counterpart for a large number of different disorders such as depression, anxiety disorders, post-traumatic stress disorder, insomnia, eating disorders, and several somatic conditions (22, 23). The modules in an ICBT treatment are often paired with some form of therapist support, usually via e-mail or telephone, but unguided treatments are also common. Guided ICBT seems to have higher rates of adherence to treatment (24, 25), and some evidence points to it being slightly more effective than unguided treatments, although more research is needed (19, 26). In a recent meta-analysis of ICBT treatment for depression, the combination of telephone and e-mail support was found to perform better than other types of minimal guidance (15). There are several potential benefits of delivering treatment over the Internet. Patients can interact with the treatment when and where they want as long as they have Internet access, it can potentially reach patients that would have otherwise not sought treatment, and it is less time-consuming for therapists and probably cost-effective (27, 28). The possibility of reaching those that would otherwise have not sought treatment is particularly interesting when it comes to GD as only 21% of problem gamblers seek treatment globally (29).

During the past few years, there has been a surge in published clinical trials of online treatments for gambling problems and GD, both CBT-based and other forms. In a recent systematic review (30), 22 studies of online treatments for gambling problems or GD were identified, of which seven were ICBT treatments. In the majority of the 22 studies, the treatment was compared to some form of control group. However, in only four of these, the experimental treatment was found effective compared to the control group, and in all these cases, the control was a waiting list (31–34). Most studies found a positive within-group effect (30). In a meta-analysis of 13 studies of online interventions for problem gambling or GD, a range of online interventions were included, with ICBT once again being the most common approach. When pooling results, Internet-delivered treatments were found to be effective for gambling symptoms. The effects were maintained and even slightly increased during follow-up assessments. However, the studies utilized a control group in only four cases. As expected, the effects in these studies were much lower (*g* = 0.47) than in the studies without a control group (*g* = 1.23). Another interesting finding is that the seven treatments including therapist support showed markedly higher effects (*g* = 1.23) than those without (*g* = 0.39) (35). In summary, so far there has been a lack of effect for any online treatment of problem gambling of GD when compared to an active control group. However, so far, most studies have been feasibility or pilot trials and not primarily designed to evaluate the effect of treatment (30).

Based on the available evidence, the most promising online treatment for GD is therapist-guided ICBT. CBT is considered the overall most promising treatment form for GD (10–12), ICBT treatments have shown promising effects in pilot trials (32, 36, 37), and ICBT treatments with therapist support have greater adherence (24, 25) and might be more effective than those without both for other psychiatric disorders (19, 26) and for GD specifically (35). Even so, there is only one published RCT comparing an ICBT-based, therapist-assisted program with an active control group (38). In that study, no difference was found between the treatment and a control group, consisting of participants monitoring their gambling expenditures, regarding the reduction of gambling symptoms at post-treatment. The study was, however, a feasibility trial with a limited number of participants (*n* = 43). There is clearly a lack of well-powered studies rigorously evaluating the potential effect of such a treatment compared to an active control. The aim of this study was to investigate the effect of a therapist-assisted ICBT treatment for GD compared to an active control treatment in a randomized controlled trial. Our primary hypothesis was that the ICBT treatment would prove significantly more effective at reducing symptoms of GD than the control treatment. Secondarily, we hypothesized that the ICBT treatment would be more effective at reducing other gambling-related outcomes of amount of money bet/week and time spent gambling/week, reducing co-occurring symptoms of depression and anxiety, reducing gambling-related cognitive distortions, and increasing self-rated quality of life. In addition, we wanted to explore possible differences in treatment credibility and patient-rated therapeutic alliance between groups as possible differences could potentially affect treatment outcomes. Finally, adverse events were explored in both treatments to assess the tolerability of the treatments.

## Materials and methods

### Participants and study design

The study is a parallel group randomized controlled trial (RCT) and is reported in accordance with the Consolidated Standards of Reporting Trials (CONSORT) guidelines for reporting parallel group randomized trials (39). It was prospectively registered in the International Standard Randomized Controlled Trial Number (ISRCTN) registry, ID: ISRCTN38692394. The trial was approved by the Regional Ethics Board in Gothenburg, Sweden (2018-08-15/631-18). Participants were recruited at the Clinic of Gambling Addiction and Screen Health at Sahlgrenska University Hospital, Department of Addiction and Dependency, in Gothenburg, Sweden. The clinic is an outpatient facility offering treatment for pathological gambling and gaming addiction and has an uptake of 1.7 million inhabitants. To be eligible for the study, participants had to (a) be between 18 and 75 years old, (b) meet the DSM-5 criteria for GD (3) as assessed by the *Structured Clinical Interview for Gambling Disorder (SCI-GD)* (40), (c) have access to the Internet and a device to interact with the treatment (computer, smartphone, tablet), and (d) be able to read and write Swedish fluently. Participants were excluded if they (e) had somatic or psychiatric conditions that contraindicated treatment or severely hindered treatment participation (e.g., ongoing psychotic, manic, or hypomanic episode, or a developmental disorder causing severe disability), (f) increased risk of suicide based on assessment during a diagnostic interview with *The Mini-International Neuropsychiatric Interview (M.I.N.I*.) (41), (g) were currently in another ongoing psychological treatment for GD (such as CBT or MI focused on gambling behavior), (h) had started medication for a psychiatric condition during the last 3 weeks (if participants had been on medication for longer than 3 weeks they were not excluded), or (i) had plans to start another treatment (psychotherapy or medication) for their GD during the course of the 8-week treatment period in this study.

### Procedure

**Figure 1:**
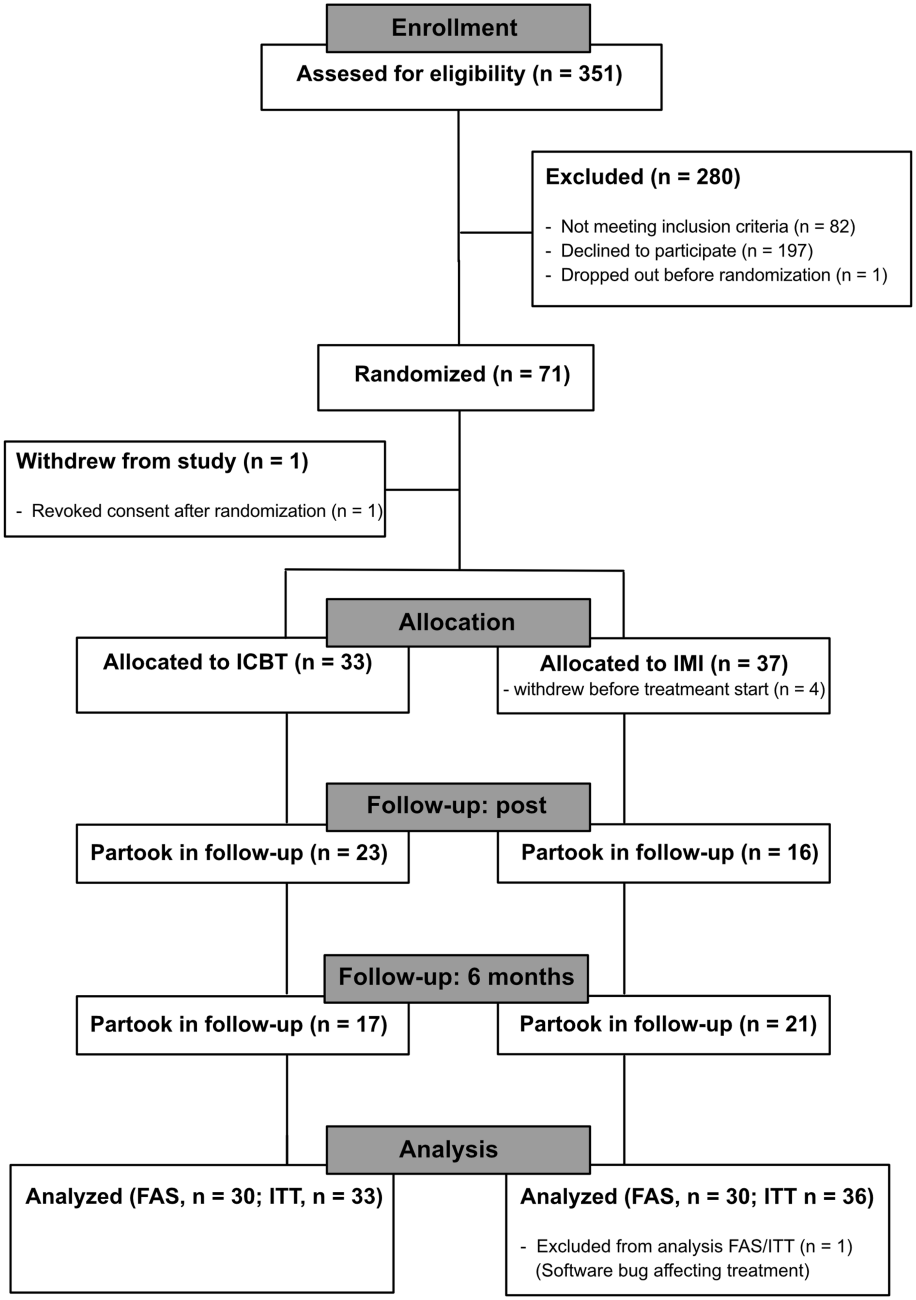
CONSORT flowchart. ICBT, internet-based cognitive behavioral therapy. FAS, full analysis sample. ITT, intention to treat population.

Everyone seeking treatment for gambling problems at the Clinic for Gambling Addiction and Screen Health, either by referral or self-referral, with a first visit at the clinic between May 2019 and November 2022 and who were considered eligible were asked about participation in the study. The trial ended when the target number of participants (at least 32 in each treatment group) was met and the last follow-up measure was collected in June 2023. The first visit was conducted by a psychiatric nurse, social worker, or psychologist at the clinic and included an anamnestic interview, as well as a structured clinical interview (SCI-GD). Owing to this, some were immediately recognized as not fulfilling the inclusion criteria during the first visit, i.e., by not fulfilling the criteria for a GD diagnosis or by having other psychiatric conditions contraindicating treatment. These were not asked about participation. All eligible that declined participation were offered the standard treatment at the clinic, consisting of CBT in individual or group format. After consent was given, a research assistant contacted the participant and conducted the M.I.N.I. clinical interview as well as an additional interview about exclusion criteria. All research assistants were psychologists in training and were supervised by the first author. Clinical psychologists at the clinic discussed all potential participants for inclusion. The M.I.N.I., SCI-GD, anamnestic interview, and additional interview about exclusion criteria, together with any prior established diagnoses from other healthcare services described in the referral, were used to assess inclusion and exclusion criteria. Before final inclusion, a research assistant once again contacted the participants over the phone to make sure that all points of the written consent were understood, and if not give the participants the chance to retract their consent. Participants were then randomized to either the ICBT treatment or a control treatment consisting of limited psychoeducation and motivational support using simple randomization. An independent statistician using the R software generated the sequence. An administrator then put each number in the sequence in a concealed envelope. Finally, upon the inclusion of a participant, a research assistant opened the topmost envelope in the stack and started the participant on the correct condition. During treatment, participants were blinded as to which treatment they had been randomized to. The study procedure is illustrated in Figure 1. At the end of the treatment period, all participants were contacted and interviewed by a psychologist about the need for additional treatment at the clinic. If participants expressed a need or the psychologist deemed that the treatment results were insufficient, additional treatment or follow-up was offered. Participants were also contacted by telephone and reminded to respond to questionnaires by a research assistant post-treatment and at follow-up.

### Interventions

Participants accessed both interventions via a secure online platform. Each intervention consisted of eight modules given over 8 weeks. Participants received a new module each week. In both interventions, participants once a week also received telephone support from their therapist for a maximum of 15 min. When a new module was started, they received a short text message reminding them of the new module. As the study was done in a clinical setting, treatments sometimes coincided with holidays. In some cases, this meant the treatment was paused for a maximum of 2 weeks (no new modules, no telephone support) and restarted after the holiday period. In these cases, the time from start to termination was up to 2 weeks longer, but participants received the same amount of treatment (eight modules, eight telephone calls).

The ICBT intervention was first described in a 2008 article by Carlbring and Smit (32). It is based on established CBT methods used in the treatment of GD. The treatment modules include psychoeducation, motivational exercises, reflecting over reasons for gambling, exercises involving significant others in the treatment, working on economic problems, making plans to handle risk situations, and other general CBT interventions (i.e., acceptance strategies, finding alternatives to irrational beliefs, finding alternative behaviors to gambling). Each module includes written exercises on the various topics and a final reflection exercise about the work done in the module. The telephone support consisted of further discussion on important aspects of the module with a focus on CBT methods and exercises. The telephone call was also used to set up and review homework assignments for participants to work on during the week (Table 1). The content of the ICBT treatment is similar to several other ICBT treatments for GD published in recent years (34, 42, 43) both in that they all offer a wide variety of interventions and that the types of interventions are similar. There are, however, small variations. The current treatment, for instance, includes quite a lot of motivational work and provides guidance over the telephone, which is less common in Internet interventions. On the other hand, some treatments include interventions not present in the ICBT treatment used in the current study, such as imaginal exposure (34), non-disorder-specific general modules (43), or relaxation exercises (42). Although this smorgasbord approach is the most common, there is also one example of an ICBT treatment where interventions focus on a smaller number of target behaviors (44).

**Table 1:** Treatment modules of both interventions.

	ICBT^a^	IMI^b^
Module 1	Psychoeducation about GD^c^, in-depth analysis of reasons for and consequences of gambling.	Short information about GD, open ended questions about expectations on treatment.
Module 2	Analysis of economic consequences, situational analysis of gambling behaviors, alternative behaviors.	Information about GD prevalence, motivational exercise.
Module 3	Involving a significant other in treatment, motivational exercise.	Open ended questions about personal strengths.
Module 4	Setting goals, acceptance strategies, further involvement of significant others.	Exploring important values.
Module 5	Irrational beliefs, alternative thoughts, behavioral activation.	Setting goals, motivational exercise.
Module 6	Strategies for managing risk situations, strategies for managing abstinence.	Motivational questions and short information about managing economy.
Module 7	Problem-solving economic difficulties, strategies for managing economy.	Information about abstinence and motivational exercises.
Module 8	Relapse prevention, information about support groups.	Information about relapse and about support groups.

a Internet-delivered cognitive behavioral therapy.

b Internet-delivered motivational interviewing.

c Gambling disorder.

The control condition was based on a motivational interviewing (MI) framework (45) and was thus dubbed Internet-delivered motivational interviewing (IMI). It had considerably shorter modules, which did not include any CBT techniques. The modules mainly focused on general psychoeducation, i.e., about what it means to have GD, which participants could partake in voluntarily. They also included motivational exercises derived from an MI framework, i.e., reflecting over the advantages and disadvantages of changing one’s gambling behavior and exploring important values in one’s life. The psychoeducational content of the IMI condition in many cases touched on similar themes as in the ICBT intervention, such as economy or abstinence. The information was, however, generally less in-depth and was not coupled with CBT strategies or exercises. Instead, MI-style open-ended questions were used to help participants reflect themselves. The telephone support consisted of motivational support using MI methodology. No homework assignments were given in the IMI condition (Table 1).

### Therapists

Participants were treated by a total of *n* = 10 therapists, including the first author. All therapists treated participants in both conditions and were either licensed psychologists (*n* = 7) or psychologists in their first year of practical training after examination as a psychologist (*n* = 3). Therapists received education in the two interventions and individual supervision, by the first author. All therapists also underwent MI training and had a conversation recorded, coded, and assessed by an independent MI trainer. Therapists were required to have passed the assessment procedure to be qualified as a therapist in the IMI condition.

### Measures

#### Clinical interviews

The *Structured Clinical Interview for Gambling Disorder (SCI-GD)* is a semi-structured diagnostic interview developed for assessing the DSM criteria for GD. The version used in this trial is updated to reflect the nine DSM-5 criteria for GD. Meeting 4–5 criteria is considered mild GD, 6–7 moderate GD, and 8–9 severe GD (3). The SCI-GD has shown high inter-rater reliability (kappa = 1.00) and excellent test–retest reliability (*r* = 0.97) (40).

The *Mini-International Neuropsychiatric Interview (M.I.N.I.)* is a structured screening interview based on diagnostic criteria from the DSM-5 (3) and covers a wide range of psychiatric diagnoses. It has shown excellent inter-rater reliability (all kappa values over 0.75) and good test retest reliability (61% of kappa values over 0.75) (41). It is widely used in Swedish healthcare and has shown good acceptability in a Swedish clinical setting (46).

*Practical questions about participation* were an additional interview including practical questions covering the final exclusion criteria, i.e., if participants had access to the Internet, and if they recently started any psychotropic medication or had plans to start another treatment with similar content as the one studied. This interview was conducted together with the M.I.N.I.

#### Self-report questionnaires

All self-report questionnaires were administered in the same online platform as the interventions. Participants had to first answer the questionnaires each week to access the treatment. This ensured participant response as long as they were active in the treatment.

##### Primary outcome measure

The *NORC Diagnostic Screen for Gambling Disorder (NODS)* was used as the primary outcome measure. It was selected based on having previously shown to be sensitive to treatment in studies of ICBT for GD (32, 36). The NODS consists of 17 yes/no questions and is scored on a scale between 0 and 10, with higher scores translating into more gambling-related problems. It has been shown to have adequate construct validity, good internal consistency (α = 0.88) (47), and excellent test–retest reliability (*r* = 0.98) for the past-year version when administered with a 2- to 4-week interval (48). For this study, the NODS was adapted to ask about the latest 14 days. The NODS was administered to participants at their first visit (here a 30-day version of the questionnaire was used), baseline (treatment start), every 2 weeks during treatment, post-treatment, and at 6-month follow-up. Internal consistency in the study population at baseline was α = 0.91.

##### Secondary outcome measures

Time spent gambling each week and the amount of money bet each week were derived from the *Time-Line Follow Back adapted to gambling (G-TLFB)* and used as secondary outcome measures. The G-TLFB is a diary tracking frequency and duration of gambling, type of game, and money bet, won, and lost (49). It was administered at baseline, each week during treatment, post-treatment, and 6-month follow-up. In the first version of the ISRCTN trial registry, the G-TLFB was listed as a primary outcome measure. This was later changed as there should be only one primary outcome measure, and the NODS was selected for this. The NODS was also used for determining sample size.

Change in symptoms of depression was assessed using the *Patient Health Questionnaire (PHQ-9)* which was developed to assess the severity of depressive symptoms. It has shown good construct validity, good internal consistency (α > 0.86), and test–retest reliability (*r* = 0.84) (50). The PHQ-9 was administered at the first visit, baseline, each week during treatment, post-treatment, and 6-month follow-up. Internal consistency in the study population at baseline was *α* = 0.84.

The *Generalized Anxiety Disorder Assessment (GAD-7)* was used to assess changes in symptoms of anxiety. It has shown good construct validity, excellent internal consistency, and good test–retest reliability (*r* = 0.83) (51). It was administered at the first visit, baseline, every 4 weeks during treatment, post-treatment, and 6-month follow-up. Internal consistency in the study population at baseline was *α* = 0.92.

Change in cognitive distortions related to gambling was measured using the *Gambler’s belief questionnaire (GBQ)*. The GBQ has been validated in a Swedish context and has shown good construct validity and excellent internal consistency (*α* = 0.94) (52). Adequate test–retest reliability was found during the development of the English version of the instrument (*r* = 0.77) (53). The English version has also been shown to be sensitive to treatment (54). The GBQ was administered at the first visit, baseline, every 4 weeks during treatment, post-treatment, and 6-month follow-up. Internal consistency in the study population at baseline was *α* = 0.87.

The *Brunnsviken Brief Quality of life scale (BBQ)* was used to measure subjective quality of life. It has shown good construct validity, adequate internal consistency (α = 0.76), and good test–retest reliability (*r* = 0.82) (55). It was administered at the first visit, baseline, post-treatment, and 6-month follow-up. On the BBQ, a higher score is indicative of a better quality of life. Internal consistency in the study population at baseline was *α* = 0.80.

##### Alliance and treatment credibility

The *Revised short version of the Working Alliance Inventory (WAI-SR)* (56) is a revised 12-item version of the original Working Alliance Inventory (57) which is designed to measure the alliance between patient and therapist during treatment. The *Treatment Credibility Scale* is an adapted version of the Credibility Scale (58). It contains five items measuring perceived credibility and expectancy of the current treatment. Both these instruments were administered once, four weeks into treatment.

##### Adverse events

Adverse events were tracked using the 20-item short form of the *Negative Effects Questionnaire (NEQ)* (59). The NEQ short form consists of 20 yes/no questions, where participants are asked if a certain type of negative effect has occurred during treatment. If a yes answer is given, participants are asked to rate how negatively this affected them on a scale of 0–4, where 0 means “not at all” and 4 “extremely negative.” The total score ranges between 0 and 80. The NEQ was administered post-treatment.

##### Demographics

A demographic questionnaire was developed specifically for this study. This was administered at baseline (treatment start).

### Statistical analysis

The sample size was determined to demonstrate an expected difference of 2 points on the NODS between the ICBT and IMI group post-treatment. Assumptions were SD = 2.5, alpha = 0.05, and power = 80%. The assumptions for SD and change during treatment were derived from a previous study of the ICBT treatment (32). A sample size inflation of 20% was employed to account for missing data, for a final sample size of *n* = 64 randomized and starting treatment (32 per treatment group). In the ISRCTN trial registry, a larger sample size was first registered. During the course of the study, it was discovered that the original sample size calculation was faulty, and it was therefore revised with a correct calculation.

One participant decided to revoke their consent and drop out of the study after randomization and is therefore not included in any analysis. Another participant in the IMI group was excluded from analysis as a software bug caused a problem with their treatment that was unfortunately discovered first after several weeks. This bug resulted in the treatment missing most of its content, and the participant was therefore deemed to not have received the planned treatment. As this problem came to our attention during the study, we opted to include one extra participant in this group. Due to the use of a simple randomization process, this also had the effect of an extra participant being included in the ICBT group. In total, 71 participants were randomized, of which 69 were possible to include in the analysis. One revoked their consent, and four withdrew before treatment start resulting in a total of 66 participants randomized and starting treatment.

On the WAI, a number of participants (*n* = 5) had suspect answering patterns where answers on question 4 and/or 10, which has reversed scoring, were not in line with the rest of the responses (i.e., near maximum scores on alliance on all other items, and lowest possible scores on reversed questions). In these cases, the assumed correct value was imputed instead.

All analyses were made in IBM SPSS version 28.0.1.1. The statistical analysis plan was developed together with a statistician. The main analysis was performed on the full analysis sample (FAS), defined as all randomized subjects with a baseline measurement and at least one post-baseline measurement on the primary outcome measure (NODS). Of these, one was excluded due to a software bug as explained above, resulting in ICBT: *n* = 30, IMI *n* = 30 being analyzed. A sensitivity analysis was performed on the intention-to-treat (ITT) population, consisting of all randomized subjects except those excluded from analysis due to reasons explained above (ICBT: *n* = 33, IMI: *n* = 36). All tables presented in the results section are for the main analyses. The corresponding tables for the sensitivity analyses can be found in the Supplementary material.

Some participants answered their post-treatment questionnaires late. All answers 2 weeks after treatment termination were considered missing. In the FAS population, a total of *n* = 19 (ICBT *n* = 7, IMI *n* = 13) participants were lost to post-treatment assessment, and a total of *n* = 22 (ICBT *n* = 13, IMI *n* = 10) were lost to 6-month follow-up. In the ITT population, *n* = 28 (ICBT: *n* = 10, IMI *n* = 19) were lost to post-treatment assessment and *n* = 31 (ICBT: *n* = 16, IMI *n* = 16) to 6-month follow-up.

Frequencies, means, and standard deviations for demographics and baseline characteristics were calculated and reported separately for the FAS and ITT populations. Demographic variables were age, gender, place of birth, education, civil status, occupation, economic situation, smoking, duration of gambling problems, gambling disorder severity, and previous treatment. Baseline characteristics were values of the NODS, G-TLFB, PHQ-9, GAD-7, GBQ, and BBQ at treatment start. Money bet/week on the G-TLFB was originally measured in Swedish (SEK) but was recalculated to US dollars for ease of understanding. Internal consistency of the outcome measures was calculated on the FAS population. Possible significant differences between demographic and baseline variables were explored using chi-square tests and Fisher’s exact tests (when over 20% of cells had expected counts less than 5) for categorical variables and t-tests and Mann–Whitney U-tests (when not normally distributed) for continuous variables.

For the FAS population, repeated measures analysis of covariance (ANCOVA) adjusted for baseline, time, time x treatment, and time x baseline was used to analyze all primary and secondary outcomes. For the ITT population, the baseline was left as part of the outcome and not used as a covariate. Therefore, repeated measures analysis of variance (ANOVA) adjusted for time x treatment was used instead. To conduct a full ITT analysis, the outcome measures at first assessment were added to the ITT model where available (NODS, PHQ-9, GAD-7, GBQ, and BBQ) as the amount of missing data were lower at this measurement point. Missing data ranged between one and three cases depending on the variable at this assessment. Missing datapoints at first assessment in the ITT population were imputed using simple mean imputation. Mean differences in the outcome variables post-treatment and at 6-month follow-up were calculated using estimated marginal means.

To see whether there were any statistical differences in perceived working alliance (WAI), treatment credibility (TCS), and patient-rated impact of adverse events (NEQ) between the treatment groups in the FAS and ITT sample, ANOVA was used.

Data were assumed to be missing at random (MAR), and maximum likelihood estimation using the REML method was used to account for missing data. Denominator degrees of freedom were calculated using Kenward-Roger approximation. To account for the possibility of therapists affecting outcomes, a random effect for therapists was added to the model (60). However, due to convergence issues, this effect had to be dropped from the model for money bet/week, the GAD-7, the GBQ and the NEQ in both samples, and additionally from the BBQ in the FAS sample. In the repeated measures analyses, gradual simplification of the covariance matrix was applied in case of problems with convergence. This resulted in the following covariance matrixes being used for each analysis, respectively (FAS sample unstructured: NODS, GAD-7, GBQ, BBQ; FAS sample AR(1) heterogenous: PHQ-9, G-TLFB money bet/week and minutes/week; ITT sample unstructured: NODS, GAD-7, GBQ, BBQ; ITT sample AR(1) heterogenous: PHQ-9, G-TLFB money bet/week and minutes/week).

A simple Mann–Whitney U-test was used to explore possible significant differences between groups on a number of modules started by participants in the FAS population as there was no missing data and data were skewed on this variable.

The frequency in % of participants reporting negative effects and the frequency for each adverse event reported in the NEQ questionnaire were calculated in the FAS sample.

Finally, after all planned analyses had been performed, an exploratory analysis was performed on the FAS and ITT populations. The effect of time from baseline to post-treatment, and post-treatment to 6-month follow-up was analyzed for all participants in both treatment groups using a repeated measures general linear model. The FAS analysis included all measures from baseline to 6-month follow-up, while the ITT analysis also included the measurement at first assessment as described above. REML method, Kenward-Roger approximation, inclusion of random therapist effects, and gradual simplification of covariance matrixes were used as described above. Random therapist effects had to be dropped due to convergence issues for GAD-7, GBQ, and money bet/week in both samples. As the FAS sample model did not include assessments at the first visit, a paired sample t-test was used to assess whether the difference between the first assessment and baseline differed significantly in this sample.

Effect sizes were calculated by dividing the estimated effect by the observed standard deviation at baseline (61). Pearson’s correlations of observed values between baseline and post-treatment, and post-treatment to 6-month follow-up were used to calculate confidence intervals for within-group effect sizes.

## Results

### Participants

A total of 71 participants were randomized to treatment, of which three could not be reached to start treatment, one chose to withdraw before treatment start, and one withdrew from the study altogether and revoked their consent. A total of three participants in the ICBT group and six in the IMI group did not fulfill the criteria to be included in the FAS population. Finally, one participant in the IMI group was excluded from the analysis due to being affected by a software bug that severely altered the treatment. Therefore, a total of 30 participants were left in each group for the main analyses, as shown in Figure 1.

The vast majority of the 60 participants in the total sample (*n* = 49, 81.7%) were male (Table 2). The mean age was 34.0 years (SD = 9.5). Most participants were born in Sweden (*n* = 57, 95%), 49 (81.7%) had a high school degree or higher, and a majority of 40 (66.7%) were married or in a stable relationship. Most of the participants, 54 (90.0%), either worked or were students. Only 8 (13.3%) participants were smokers, and 21 (35.0%) had previously sought help for their gambling problems. There were no significant differences between the treatment groups for any of the demographic variables, except for education, where the IMI group had a significantly lower level of education than the ICBT group (*p* < 0.05). Post-treatment, a total of *n* = 23 were offered some form of additional treatment or follow-up (ICBT: individual CBT *n* = 1, group CBT *n* = 1, limited follow-up via telephone *n* = 7; IMI: individual CBT *n* = 3, relapse prevention *n* = 3, limited follow-up via telephone *n* = 8).

**Table 2:** Demographics for the full analysis sample.

	ICBT group^a^	IMI group^b^
	(*n* = 30)	(*n* = 30)
**Gender, *n* (%)**		
Female	5 (16.7)	6 (20.0)
Male	25 (83.3)	24 (80.0)
**Age, mean (SD)**	33.1 (8.2)	34.9 (10.8)
**Place of birth, *n* (%)**		
Sweden	27 (90.0)	30 (100)
Non-Nordic European country	2 (6.7)	-
Asia	1 (3.3)	-
**Education, *n* (%)** ^**c**^		
Less than high school	1 (3.3)	10 (33.3)
High school	17 (56.7)	13 (43.3)
University	12 (40.0)	7 (23.3)
**Civil status, *n* (%)**		
Married/In a stable relationship	20 (66.7)	20 (66.7)
Divorced/Separated/Widow(er)	4 (13.3)	-
Single	5 (16.7)	8 (26.7)
Other	1 (3.3)	2 (6.6)
**Occupational status, *n* (%)**		
Working/student	26 (86.7)	28 (93.3)
Sick-leave	1 (3.3)	2 (6.7)
Unemployed	1 (3.3)	-
Parental leave	1 (3.3)	-
Other	1 (3.3)	-
**Self-reported financial status, *n* (%)**		
Very bad	6 (20.0)	9 (30.0)
Bad	9 (30.0)	9 (30.0)
Neither good or bad	7 (23.3)	7 (23.3)
Good	6 (20.0)	4 (13.3)
Very good	2 (6.7)	1 (3.3)
**Duration of gambling problems, *n* (%)**		
>1 year	-	1 (3.3)
1–2 years	8 (26.7)	7 (23.3)
3–5 years	9 (30.0)	7 (23.3)
6–10 years	10 (33.3)	4 (13.3)
More than 10 years	3 (10.0)	11 (36.7)
**Gambling disorder severity, *n* (%)**		
Mild	5 (16.7)	7 (23.3)
Moderate	18 (60.0)	12 (40.0)
Severe	7 (23.3)	11 (36.7)
**Previous treatment for gambling problems, *n* (%)**		
Yes	11 (36.7)	10 (33.3)
No	19 (63.3)	20 (66.7)
**Smoker, *n* (%)**		
Yes	4 (13.3)	4 (13.3)
No	26 (86.7)	26 (86.7)

a Internet-delivered cognitive behavioral therapy.

b Internet-delivered motivational interviewing.

c Statistically significant difference between treatment groups, chi-square test, *p* < 0.05.

### Primary and secondary outcomes

No significant difference in gambling symptoms was found for the primary outcome variable NODS between the ICBT and IMI groups post-treatment (*p* = 0.821) or at 6-month follow-up (*p* = 0.254; Tables 3, 4). The NODS scores in both groups indicate no current gambling problems post-treatment [mean ± SD (range), ICBT: 0.2 ± 0.4 (0–1); IMI: 0.3 ± 1.0 (0–4)]. At the 6-month follow-up, the mean scores in both groups indicate at-risk gambling [ICBT: 1.1 ± 2.4 (0–7); IMI: 1.0 ± 2.5 (0–9)], and two participants in each group had a NODS score ≥ 5 which is the cutoff for probable GD, signifying possible relapses. There was no significant difference between treatments at baseline for the primary outcome in either population. Baseline scores for the NODS ranged between 0 and 10 in the ICBT group and 0 and 6 in the IMI group. There were also no significant differences for any of the secondary outcomes (amount of money gambled/week, minutes gambled/week, depressive symptoms, anxiety symptoms, cognitive distortions, and quality of life) between the ICBT and IMI groups post-treatment or at 6-month follow-up (Tables 3, 4). The post-treatment mean of amount bet/week in the IMI group and the 6-month follow-up mean in the ICBT group were higher than the corresponding values at baseline. This was due to a single participant in each group gambling for a large amount during a relapse. Both groups had non-clinical symptom scores at post-treatment on the PHQ-9 [mean ± SD (range), ICBT: 3.4 ± 3.9 (0–13); IMI: 1.3 ± 1.6 (0–5)] and the GAD-7 [ICBT: 2.8 ± 1.5 (0–12); IMI: 1.5 ± 2.4 (0–6)]. Quality of life as measured by the BBQ was at non-clinical levels for both groups at post-treatment [ICBT: 63.0 ± 23.6 (20–96); IMI: 67.2 ± 26.2 (6–96)]. At 6-month follow-up, the PHQ-9 showed mild depressive symptoms in the ICBT group whereas scores in the IMI group were still non-clinical [ICBT: 5.0 ± 7.1 (0–23); IMI: 3.1 ± 3.8 (0–14)]. Anxiety symptoms on the GAD-7 [ICBT: 4.1 ± 5.4 (0–18); IMI: 2.9 ± 4.0 (0–14)] and quality of life measured by the BBQ [ICBT: 57.6 ± 29.5 (0–96); IMI: 60.2 ± 24.6 (13–96)] were at non-clinical levels in both groups. There were no significant differences between treatments at baseline for any of the secondary outcomes in either population. There were likewise no significant effects for the primary and secondary outcomes in the ITT sensitivity analyses. Detailed results of the sensitivity analysis can be found in the Supplementary material (Supplementary Tables 1–3).

**Table 3:** Observed primary and secondary outcomes (full analysis sample).

Measure	Baseline^a^	2	3	4	5	6	7	8	Post	6 months
**NODS**										
ICBT^b^	1.6 (2.3)		1.1 (2.0)		0.9 (1.5)		0.5 (1.0)		0.2 (0.4)	1.1 (2.4)
IMI^c^	1.2 (2.0)		0.7 (1.7)		0.6 (1.3)		0.7 (1.4)		0.3 (1.0)	1.0 (2.5)
**Amount bet/week** ^**d**^^**,**^^**e**^										
ICBT	219.0 (674.8)	101.9 (494.0)	41.0 (203.7)	242.6 (1203.4)	0.8 (4.0)	0.7 (3.6)	5.5 (20.9)	1.5 (6.5)	2.3 (7.5)	544.7 (2245.8)
IMI	192.7 (835.3)	199.1 (603.9)	34.4 (120.8)	47.6 (223.1)	102.0 (464.3)	43.7 (198.4)	50.6 (163.5)	0.0 (0.0)	200.2 (801.0)	47.1 (118.0)
**Minutes gambled/week** ^**d**^										
ICBT	133.7 (454.1)	183.6 (839.8)	15.0 (71.9)	24.2 (107.7)	1.3 (6.3)	1.3 (6.1)	4.5 (14.7)	1.7 (7.1)	1.4 (6.4)	58.8 (242.5)
IMI	52.5 (171.2)	66.4 (198.1)	21.0 (68.4)	28.6 (134.3)	36.0 (124.9)	25.2 (87.3)	27.0 (84.8)	0.0 (0.0)	41.6 (166.3)	42.3 (123.2)
**PHQ-9**										
ICBT	5.7 (3.5)	5.2 (4.8)	4.2 (4.4)	4.4 (4.9)	4.4 (4.6)	3.2 (3.2)	3.2 (3.4)	2.7 (3.3)	3.4 (3.9)	5.0 (7.1)
IMI	7.0 (5.4)	5.4 (4.6)	4.8 (3.6)	4.0 (3.9)	3.4 (3.3)	3.0 (2.4)	2.6 (2.4)	2.3 (2.1)	1.3 (1.6)	3.1 (3.8)
**GAD-7**										
ICBT	5.7 (5.0)				3.7 (4.8)				2.8 (4.1)	4.1 (5.4)
IMI	6.0 (5.2)				3.1 (3.3)				1.5 (2.2)	2.9 (4.0)
**GBQ**										
ICBT	68.0 (24.3)				57.8 (25.6)				54.3 (24.1)	45.5 (24.0)
IMI	67.7 (23.6)				54.3 (28.1)				42.1 (24.9)	36.0 (18.5)
**BBQ**										
ICBT	51.7 (21.2)								63.0 (23.6)	57.6 (29.5)
IMI	52.4 (15.8)								67.2 (26.2)	60.2 (24.6)

Data are shown as mean (standard deviation).

a Treatment start, followed by each week in treatment, post-treatment, and 6-month follow-up.

b Internet-delivered cognitive behavioral therapy.

c Internet-delivered motivational interviewing.

d Measured by the gambling timeline follow back.

e Presented in US $. originally stated in Swedish (SEK; Exchange rate 1 June 2023).

**Table 4:** Estimated mean differences post-treatment and at 6-month follow-up between the ICBT^a^ and the IMI^b^ treatment in the full analysis sample.

Measure	Post-treatment^c^	*p*-value^d^	6-month follow-up^e^	*p*-value^f^
NODS	0 [−0.4 – 0.3]	0.821	−0.9 [−2.6 – 0.7]	0.254
Amount bet/week^g^^,^^h^	186.2 [−57.6 – 430.1]	0.129	−553.6 [−1655.5 – 548.2]	0.313
Minutes gambled/week^g^	23.5 [−15.3 – 62.2]	0.226	−45.7 [−189.7 – 98.2]	0.522
PHQ-9	−1 [−2.4 – 0.5]	0.197	−1.6 [−5.5 – 2.3]	0.408
GAD-7	−1.1 [−3.0 – 0.8]	0.254	−0.8 [−3.6 – 2.1]	0.578
GBQ	−8.4 [−19.7 – 2.9]	0.141	−7 [−20.2 – 6.2]	0.289
BBQ	−0.1 [−10.4 – 10.2]	0.983	−0.1 [−12.0 – 11.8]	0.985

Data are shown as mean (95% confidence interval [CI]).

a Internet-delivered cognitive behavioral therapy.

b Internet-delivered motivational interviewing.

c Model estimated mean difference post-treatment. Positive values indicate a lower value in the ICBT treatment arm.

d Calculated using estimated mean differences post-treatment.

e Model estimated mean difference at 6-month follow-up. Positive values indicate a lower value in the ICBT treatment arm.

f Calculated using estimated mean differences post-treatment.

g Measured by the gambling timeline follow back.

h Presented in US $. originally stated in Swedish (SEK; Exchange rate 1 June 2023).

### Treatment credibility, alliance, and treatment retention

Alliance and treatment credibility (measured by WAI-SR and TCS) were scored highly in both groups. The ICBT group had a mean score on the TCS of 40.3 (SD = 6.9). The corresponding score for the IMI group was 36.4 ± 11.4. The model estimated mean difference of 3.8 [95% CI, −1.3–9.0] was not statistically significant (*p* = 0.138). The corresponding scores for the WAI-SR were 75.8 ± 7.9 for ICBT and 72.3 ± 11.1 for IMI. Moreover, the model estimated mean difference of 3.7 [95% CI, −1.3–8.6] was not statistically significant (*p* = 0.142). The ITT sensitivity analysis yielded the same results, with no significant differences found between treatments (estimated mean difference [95% CI], TCS: 4.1 [−1.0–9.1] *p* = 0.112; WAI: 3.8 [−1.1–8.7] *p* = 0.128). The mean number of treatment modules started (max 8) was 7.5 ± 1.3 in the ICBT group and 7.0 ± 1.8 in the IMI group, which was not a statistically significant difference (*p* = 0.223). A total of 24 participants (80%) in the ICBT group and 20 (67%) in the IMI group stayed in treatment until the final module.

### Adverse events

The NEQ was completed by 24 of the 30 participants in the ICBT group and 19 of 30 in the IMI group in the FAS population. Of the 24 completing the questionnaire in the ICBT group, 13 (54.2%) reported some type of adverse event. The following adverse events were reported in the ICBT group: felt under more stress (4/24), experienced more anxiety (5/24), felt more worried (2/24), experienced more unpleasant feelings (2/24), unpleasant memories resurfaced (8/24), afraid that other people would find out about the treatment (3/24), feeling ashamed in front of other people due to going in treatment (2/24), stopped thinking things could get better (1/24), did not always understand the treatment (1/24), did not have confidence in the treatment (1/24), felt that the treatment did not produce any results (1/24), and tiredness (1/24).

Of the 19 participants completing the questionnaire in the IMI group, 10 (52.6%) reported some type of adverse event. The following types of adverse events were reported: sleeping problems (1/19), felt under more stress (2/19), experienced more anxiety (3/19), felt more worried (1/19), experienced more hopelessness (3/19), experienced more unpleasant feelings (1/19), unpleasant memories resurfaced (4/19), afraid that other people would find out about the treatment (3/19), feeling ashamed in front of other people due to going in treatment (2/19), thinking that the issue one was seeking help for could not be made any better (2/19), did not always understand the treatment (4/19), did not always understand the therapist (1/19), did not have confidence in the treatment (2/19), and felt that the treatment did not produce any results (3/19).

Patient-related impact of adverse events measured by the NEQ total score was low in both groups. The ICBT group had a mean score of 1.6 (SD = 2.2). The corresponding score for the IMI group was 2.2 ± 4.6. The model estimated mean difference of 0.5 [95% CI, −1.6–2.7] was not statistically significant (*p* = 0.620). The ITT sensitivity analysis yielded the exact same result for the NEQ.

### Exploratory analyses—effect of time during treatment

As no differences were found between the treatment groups on any of the outcome measures, exploratory analyses were performed to study the effect of time (baseline to post-treatment, and post-treatment to 6-month follow-up) on all outcome measures during treatment on the total population in the FAS sample (*n* = 60) with sensitivity analyses performed on the total ITT sample (*n* = 69). Significant effects ranging from medium to large (*d* = 0.52–0.89) were found between baseline and post-treatment for the NODS, PHQ-9, GAD-7, GBQ, and BBQ (Tables 5, 6). Significant effects were also found between post-treatment and 6-month follow-up for the NODS, PHQ-9, and GAD-7. These effects ranged from small to medium (d = 0.30–0.59), and all indicated increased symptoms compared to post-treatment. The results were replicated in the ITT sensitivity analysis and can be found in the Supplementary material (Supplementary Tables 4,5). The total sample score for the NODS at the 6-month follow-up indicated at-risk gambling 1.1 (SD = 2.4) while the scores for PHQ-9 (4.0 ± 5.6) and the GAD-7 (3.4 ± 4.7) indicated non-clinical symptoms. Mean scores and standard deviations for first visits were also calculated for descriptive purposes. The mean NODS score during first visit was 4.8 (SD = 3.0) which was notably higher than the baseline score (1.4 ± 2.1) (Table 5), and this difference was significant (*p* < 0.001). Somewhat worse scores (higher for the PHQ-9, GAD-7, and GBQ and lower for the BBQ) could be found at the first visit compared to the baseline for all secondary outcomes measured at the first visit (Table 5).

**Table 5:** Observed values at the first visit, baseline, post-treatment, and 6-month follow-up for the total full analysis sample.

Measure	First visit	Baseline	Post	6 months
NODS^a^	4.8 (3.0)	1.4 (2.1)	0.2 (0.7)	1.1 (2.4)
Amount bet/week^b^^,^^c^	-	205.4 (755.2)	85.6 (519.5)	275.7 (1520.6)
Minutes gambled/week^b^	-	91.6 (337.5)	18.3 (107.9)	49.9 (185.0)
PHQ-9	9.5 (5.7)	6.4 (4.6)	2.5 (3.3)	4.0 (5.6)
GAD-7	7.6 (5.3)	5.9 (5.1)	2.2 (3.5)	3.4 (4.7)
GBQ	72.3 (20.2)	67.5 (23.7)	49.0 (24.9)	40.4 (21.4)
BBQ	41.2 (21.4)	52.1 (18.5)	64.9 (24.5)	59.0 (26.6)

Data are shown as mean (standard deviation).

a A significant difference was found between first visit and baseline, *p* < 0.001.

b Measured by the gambling timeline follow back.

c Presented in US $. originally stated in Swedish (SEK; Exchange rate 1 June 2023).

**Table 6:** Estimated mean effects of time between baseline to post-treatment and post-treatment to 6-month follow-up in the total full analysis sample.

Measure	Baseline – post^a^	*p*-value^b^	Effect size^c^	Post – 6 months^d^	*p*-value^e^	Effect size^f^
NODS	1.1 [−0.5 – 1.6]	< 0.001	0.52 [−1.15 – 2.02]	−0.9 [−1.8 – 0.0]	0.049	0.42 [−1.74 – 2.43]
Amount bet/week^g^^,^^h^	133.3 [−134.2 – 400.8]	0.325	N/A	−179.6 [−699.6 – 340.5]	0.489	N/A
Minutes gambled/week^g^	74.9 [−21.2 – 171.0]	0.124	N/A	−29.7 [−100.4 – 41.0]	0.398	N/A
PHQ-9	4.1 [2.6 – 5.5]	< 0.001	0.89 [−1.07 – 2.65]	−2.7 [−4.3 – −1.2]	0.001	0.59 [−0.82 – 1.86]
GAD-7	3.5 [2.2 – 4.8]	< 0.001	0.69 [−1.20 – 2.38]	−1.5 [−2.9 – −0.2]	0.026	0.30 [−0.99 – 1.48]
GBQ	20.0 [14.2 – 25.7]	< 0.001	0.84 [−0.73 – 2.29]	7.1 [−2.4 – 16.5]	0.139	N/A
BBQ^i^	−11.1 [−16.1 – −6.2]	< 0.001	0.60 [−0.61 – 1.70]	5.9 [−0.3–12.2]	0.062	N/A

Data are shown as mean (95% confidence interval [CI]).

a Model estimated mean difference with 95% confidence intervals between baseline and post-treatment for the total sample. Positive values indicate a reduction from baseline.

b Calculated for baseline – post-effect.

c Effect size with 95% confidence intervals calculated between baseline and post-treatment for significant effects.

d Model estimated mean difference with 95% confidence intervals between post-treatment and 6-month follow-up for the total sample. Positive values indicate a reduction from baseline.

e Calculated for post – 6-month effect.

f Effect size with 95% confidence intervals calculated between post-treatment and 6-month follow-up for significant effects.

g Measured by the gambling timeline follow back.

h Presented in US $. originally stated in Swedish (SEK; exchange rate 1 June 2023).

i Higher scores indicate better quality of life on the BBQ.

## Discussion

This parallel group single-blind randomized controlled trial compared the effect of an Internet-delivered therapist-assisted CBT-based treatment of GD with a control treatment. When comparing treatment groups, we found no difference in GD symptoms post-treatment or at 6-month follow-up. Furthermore, we did not find any difference between the treatment groups on the amount of money bet and time spent gambling post-treatment or at follow-up. Similarly, no significant group differences were found regarding depressive symptoms, anxiety symptoms, gambling-related cognitive distortions, and quality of life. However, when both groups were combined and analyzed as a total sample, we found a positive effect of time on GD symptoms between the start of treatment to post-treatment. In further analyses of the effect of time on the total sample, we found that depressive symptoms, anxiety symptoms, gambling-related cognitive distortions, and quality of life had also improved over time. However, for GD symptoms, anxiety, and depression, there was a deterioration between post-treatment and 6-month follow-up, with small effects for GD and anxiety and a moderate effect for depression. No effect of time was found for the gambling diary. Both treatments were found to be highly credible, the therapeutic alliance was scored highly in both groups, and the mean numbers of completed modules were also high, with no difference found between groups. Finally, over 50% of participants completing questionnaires post-treatment, in both groups, reported some type of adverse event. The total self-rated impact of adverse events was low, and there was no difference between groups.

There might be several reasons for not observing an effect for the primary outcome between treatments. First, based on previous studies using the same CBT treatment and NODS as the primary outcome measure, we assumed that participants would have a relatively high baseline score (baseline mean scores were 8.21 (32) and 8.1 (36), respectively). Post-treatment means on the NODS for these studies were 1.97 (32) and 1.8 (36). Based on this, an assumption was made that a 2-point difference between ICBT and IMI groups was a likely outcome and power was calculated from this assumption. The baseline mean scores in our study were, however, much lower (ICBT: 1.6; IMI: 1.2), and a 2-point difference was not mathematically possible. Therefore, the study was not sufficiently powered to detect a possible between-group difference on the NODS.

Second, both the ICBT and the IMI group had a NODS score close to 0 (ICBT: 0.2; IMI: 0.3) post-treatment, meaning they had almost no GD-related symptoms. It could be that both treatments were effective in reducing gambling symptoms. The IMI condition was more limited in content and lacked CBT elements, but it might be that receiving MI telephone support paired with psychoeducation was equally effective as a structured CBT treatment. In addition, there has been an accumulation of evidence in later years that low-intensity online interventions might be as effective in reducing gambling symptoms as more intensive programs. As was described in the introduction, a meta-analysis from 2021 (35) found that the effect on gambling symptoms was lower (*g* = 0.47) in the nine studies that had some form of control group, active or waitlist, compared to the six studies without any control group (*g* = 1.23). Furthermore, in a 2022 systematic review (30) of 22 studies on online interventions for gambling disorder, only four studies found a difference between the experimental condition and the control group on gambling symptoms, and in all these cases, the effect was seen in studies using a waitlist control and not an active condition (31–34). In studies where the experimental condition instead was compared to a control group receiving some form of low-intensity but active intervention [e-mail counseling (33); monitoring, feedback, and support (34); weekly log of gambling expenditures (38); personalized normalized feedback (62)], no difference between the groups was found. It might be that a low-intensity treatment is enough for most gamblers entering treatment online. In our study, there was an effect of time on gambling symptoms during the course of treatment when both groups were combined, which might indicate that both treatments were effective. However, it is not possible to fully draw such a conclusion based on the lack of waitlist control.

Third, it is possible that the treatments in our study were too similar. The IMI treatment, in comparison with the ICBT treatment, lacked CBT elements and had less content. It did, however, have weekly phone calls where MI methodology was used. In MI, it is possible for therapists to give advice if participants ask for it (45), and as the therapists were all trained in both CBT and MI, it might be that the advice given at times was based on the therapist’s knowledge of effective CBT strategies. This might have made the treatments more similar than intended, even though no structured CBT content or homework assignments were used.

Fourth, participants in both treatments rated treatment credibility and therapist alliance equally high. Patient expectancy of a positive outcome has been identified as a possible contributor to the effects of psychotherapy (63) with pre-treatment or early treatment outcome expectancy showing a small but significant association with treatment outcome (64). It is possible that the high perceived credibility and alliance in both treatments resulted in comparable expectancy effects, at least partially explaining the similar results.

Finally, it might be that parts of the procedure or process not related to the treatments themselves were enough to exact change in participants’ gambling behaviors. It is not uncommon that RCTs with psychosocial interventions fail to demonstrate effectiveness when compared to a control condition (65). Several reasons, of which some are discussed above, can be seen for this, such as a potentially good treatment but too little of it, lack of treatment adherence, or lacking statistical power (65). However, other possible reasons are that integral parts of the RCT, such as assessments, monitoring procedures (answering questionnaires), and just being observed as part of a research study, might all result in behavior change (66–68). In the current study, a process of change might have started well before treatment was initiated and continued apace during treatment. This is supported by examining the mean NODS score at the first visit (total sample: 4.8) which was substantially higher than the mean baseline score (total sample: 1.4). As this study was set in ordinary clinical practice, there was usually a wait of at least about 2–4 weeks between first visit and treatment start for practical reasons. This fact, coupled with that the NODS measurement was taken at the first visit, makes it possible to observe change already before treatment start. It might be that just the initial assessment at the clinic coupled with continuous monitoring was enough to start and maintain a process of change. Such effects might even be especially potent in GD. Very brief interventions such as personalized feedback (69) and one-session minimal interventions (70) have been shown to have treatment effects in GD, implying it is a condition where change sometimes can occur with minimal treatment. This is interesting as other studies comparing an online intervention to control also include some form of assessment procedure but only measure symptoms at baseline (33, 34, 38, 62, 71, 72). If such an effect can be seen just by undergoing an assessment procedure, it is possible that the effects seen in other comparable studies can also be partially explained by the effect of an initial assessment. The effects of assessment and monitoring could further explain why it has been hard to find differences between treatments of differing intensity. However, it should be noted that the assessment procedure in this study entailed a first face-to-face visit at the clinic and was generally more extensive than in other listed studies. It is also possible that the change occurring between assessment and treatment start was at least partly due to outcome expectance effects as detailed above. Although expectancy effects are not particularly explored in the treatment of GD, it is possible that attending the assessment procedure generated a positive effect on outcome expectancy, which in turn might partly be responsible for an early change in gambling symptoms.

It should also be noted that there is some uncertainty to the comparison of NODS scores as different versions of the NODS were used (past 30 days at the first visit, past 14 days at baseline). This means some of the changes seen between the assessment at the first visit and baseline could have started the weeks before the first visit to the clinic, but not indicated in NODS 30 days, as its longer timeframe makes it less sensitive to recent changes.

No difference was found between treatments, or over time during treatment, in the total sample for the secondary outcomes from the gambling diary (G-TLFB) of amount of money bet per week and time spent gambling per week. This lack of change over time might be due to low values already at baseline, paired with a large variability in scores. As abstinence from gambling gives scores of zero, while a setback for a few participants might give large scores at certain time points, there is an innate variability in this type of measurement. This can be seen when studying the IMI group, where both the amount bet per week and time spent gambling per week are down to zero at week 7 and then back to baseline scores at week 8 (with large standard deviations).

There was also no difference between treatments for the other secondary outcomes. Once again, baseline scores were relatively better than at the first visit, although not as much as for the NODS (PHQ-9, GAD-7, and GBQ all had higher scores at the first visit, and for the BBQ the score was lower at the first visit, but here a low score is worse). This might be explained by the same reasons given for the primary outcome, i.e., both treatments being equally effective, expectancy effects, or change occurring due to factors unrelated to the treatment such as the assessment and monitoring procedures. Indeed, if gambling symptoms change over time, it is not surprising that symptoms of depression and anxiety, and quality of life change as well. What is more surprising is that there was no difference between treatments regarding gambling-related cognitive distortions. The CBT treatment had a module (module 5) specifically addressing such thoughts, while the IMI treatment lacked any such content. Nevertheless, there was a change over time regarding these distortions when both groups were combined. It might be that the act of being abstinent from gambling alone gave participants a new perspective on, and thereby exacted change on, these cognitive distortions.

The frequency of participants experiencing some type of adverse event of those completing the NEQ was in line with the 50.9% found in the original study exploring the psychometric properties of the NEQ (59). The total self-rated negative impact of these events was low in both groups, and no difference was found between groups. This indicates that both treatments were equally tolerable. In addition, both treatments were found highly credible, alliance scores were rated highly, and retention rates were fairly high. This also indicates that participants found both treatments tolerable. There is, however, a possibility of bias due to missing data. The NEQ was only administered post-treatment and as such participants that might have withdrawn due to not finding the treatment tolerable were less likely to respond to the NEQ. Similarly, as the TCS and WAI were administered four weeks into treatment, those that might have withdrawn due to finding the treatment uncredible were less likely to have responded to these questionnaires, although for the TCS and wAI the amount of missing data were low. Overall, as participants in both treatments had almost no self-rated GD symptoms post-treatment, the positive effect of treatment likely outweighs the negative effects. The most commonly reported adverse event was that unpleasant memories resurfaced. This is not surprising as spending time thinking and talking about gambling problems might remind participants of problems caused by gambling behavior.

In addition to post-treatment data, we also included a 6-month follow-up. We saw no differences in the NODS score at the 6-month follow-up between groups. However, the 6-month follow-up indicated at-risk gambling in both groups. In addition, when analyzing the effect of time in the total sample, we found a deterioration between post-treatment and 6-month follow-up on the NODS, PHQ-9, and GAD-7, although the effects were small to moderate. Taking a closer look at the data, we saw that two participants in each group had experienced a relapse. It is highly likely that these relapses explain a large part of the deterioration seen at the group level. One explanation for relapses might be that individuals have deteriorated regarding symptoms of depression and anxiety. Perhaps what we see here is a reaction of stress trying to balance negative emotions without gambling and that relapse is once again used as a way to cope with these emotions. It is also not uncommon with relapse in GD (73). It is not possible to know whether the rate of relapse would have been even higher without treatment, and only further long-term follow-up can determine whether participants will continue to deteriorate or not. Interestingly, the effects regarding cognitive distortions and quality of life were maintained at 6 months.

In general, the findings of this study, together with other recent comparable studies, indicate that online treatments might be helpful for GD but that treatments with lower intensity or in a non-CBT format might be equally effective as more intensive CBT treatments. There is also evidence that treatments with therapist guidance perform better than unguided treatments (19, 26, 35). Low-intensity, therapist-guided online treatments might thus be a cost-effective way to treat GD. However, this does not necessarily mean this approach works for everyone with GD, and quite possibly a subset of those with GD need more intensive interventions such as face-to-face CBT. In the current study, the patients could voluntarily choose to participate. If they did not want to participate, they could instead receive face-to-face or group-based CBT. It is therefore possible that a self-selection has occurred where those with less severe problems or greater self-regulation chose to participate. Indeed, some evidence points to this as a previous study on a sample from the same clinic had a higher frequency of participants with severe GD (74). The fact that many potential participants declined participation might also point to this. It is also interesting that change seemed to at least partially occur before treatment start. If gambling symptoms are this susceptible to intervention, it is possible that some of the changes seen in treatment studies are caused by the effect of assessment and monitoring alone. It might be that the effect of assessment and monitoring is particularly strong when it comes to symptoms of GD, and this could also explain the lack of effect seen between treatment groups in numerous trials of online interventions for GD.

Since we only have 6-month follow-up data yet in this study, it is not possible to say if most participants will continue to show low symptoms of GD, although the same ICBT program that was used in the current study has previously shown sustained within-group effects up to 36 months post-treatment (32, 36). It is possible that a difference might occur in relapse rates between the ICBT and IMI treatment over longer-term follow-up. An assessment of treatment efficacy should also depend on whether the results are consistent over time or not. We therefore intend to make further follow-up assessments at 12 and 24 months after treatment end.

This RCT had both strengths and limitations. The strengths were a rigorous design following CONSORT guidelines and utilizing an active control group receiving a treatment of comparable format. Furthermore, the experimental group received an ICBT treatment that had been evaluated in previous studies with positive effects. The therapists were trained in both CBT techniques and MI and had undergone an MI coding procedure to guarantee proficiency. The credibility of treatments and therapeutic alliance were also measured, and both treatments proved to be credible and rendered a positive alliance with participants and had a similar retention rate. Another strength was that gambling symptoms were measured at the first visit, which made it possible to discover that change started well before treatment start. Finally, the study was conducted in the clinic and thus explored the effect of treatment in a real clinical sample.

The main limitation was that based on previous studies, faulty assumptions were made regarding possible between-group differences and likely baseline scores. Based on RCTs that were published after the conception of this study (33, 38, 62), other assumptions would have possibly been made resulting in a better-powered study. Another possible limitation was that no inclusion criteria were used regarding the level of gambling symptoms at treatment start. This resulted in many participants having minor to no symptoms pre-treatment. A level of symptoms set for inclusion might have explored the effect of treatments on symptomatic gamblers in a better way. On the other hand, this was a representative treatment population presenting at a clinic, and narrowing down the criteria thus would have rendered it so that only a select few would have been eligible to participate. A further limitation was that no toxicological screening was carried out, and no specific exclusion criteria were set regarding co-morbid alcohol or substance use disorder. The reason for not excluding these participants was to ensure that the sample would be as close to a true clinical population as possible. However, due to the high co-morbidity of GD with alcohol and substance use disorder, this might potentially have affected the participant’s ability to interact with the treatment. Another limitation was that symptoms of both GD and co-morbid disorders were only assessed using self-report questionnaires and were not corroborated with diagnostic interviews. Finally, the fact that a large number of potential participants declined may have led to self-selection, which can potentially have caused bias.

One must exact caution to generalize the findings of this study, based on previously discussed possible effects of self-selection and the fact that the population was largely culturally homogeneous. Nevertheless, this study combined with other previously discussed studies of online interventions points to some interesting venues for future research. First, the effect of low-intensity online interventions for GD should be further explored. Although these might not work for everyone, they might be a cost-effective way to treat GD for at least a subset of the population. Based on previous research, these interventions should include some form of therapist guidance but could be limited in scope—perhaps even more so than the control treatment in this study. Second, the effect of treatment over time needs to be further explored. It is still possible that treatments of higher intensity or using tools from CBT are better at sustaining treatment effects over time. It is important to continue studying the lasting effects of online treatments regarding both high-intensity CBT treatments and more low-intensity formats using long-term follow-ups. Third, further analysis as to who benefits from what treatment should be made. It is possible that depending on GD severity, comorbidities, social factors, etc., treatments might have different effects. It might be that online treatments or treatments of lower intensity are useful for a subset of patients while some need more intensive or face-to-face treatment. Further knowledge about who benefits from what treatment could help in treatment planning, ensuring everyone gets the treatment they need, without at the same time using more resources than needed. This could be achieved by studying the moderating effects of other factors on treatment results. Finally, the effect of assessment and monitoring in treatment trials of GD should be further explored. If assessment in itself can exact change in the magnitude alluded to in the present study, this needs to be taken into account when designing treatment studies for GD—to ensure the integrity of the results of future trials. In conclusion, more research is needed on low-intensity interventions for GD, both Internet-delivered and otherwise, as this approach might be both cost-effective and have the potential to reach more individuals with GD.

## Data availability statement

The raw data supporting the conclusions of this article will be made available by the authors, without undue reservation.

## Ethics statement

The studies involving humans were approved by Regional Ethics Board in Gothenburg, Sweden (2018-08-15/631-18). The studies were conducted in accordance with the local legislation and institutional requirements. The participants provided their written informed consent to participate in this study.

## Author contributions

MM and AG contributed to conception and design of the study. FS developed the control treatment. MM, JM, DN, HS, JR, SL, AL, and JP contributed to data acquisition (acting as therapists in the study). MM performed the statistical analysis and wrote the first draft of the manuscript. AG wrote sections of the manuscript. All authors contributed to the article and approved the submitted version.
