# Supplementary material for: Internet-delivered therapist-assisted cognitive behavioral therapy for gambling disorder: a randomized controlled trial
Source: Front Psychiatry. 2023 Dec 11;14:1243826. doi: 10.3389/fpsyt.2023.1243826 (PMC10749366; doi:10.3389/fpsyt.2023.1243826)
Supplement: Supplementary file 1 [file Table_1.DOCX]

**Supplemental Table 1.** Demographics for the Intention To Treat sample.

|  | ICBT group^1^  (n=33) | IMI group^2^  (n=36)^3^ |
| --- | --- | --- |
| **Gender, n (%)**  Female  Male | 5 (15.2)  28 (84.8) | 6 (18.8)  26 (81.3) |
| **Age, mean (SD)** | 32.9 (8.0) | 34.9 (10.6) |
|  |  |  |
| **Place of birth, n (%)**  Sweden  Non-nordic European country  Asia | 29 (87.9)  2 (6.1)  2 (6.1) | 32 (100)  -  - |
| **Education, n (%)^3^** |  |  |
| Less than high school | 1 (3.0) | 10 (31.3) |
| High school | 18 (54.5) | 15 (46.9) |
| University  Other | 13 (39.4)  1 (3.0) | 7 (21.9)  - |
|  |  |  |
| **Civil status, n (%)**  Married/In a stable relationship  Divorced/Separated/Widow(er)  Single  Other | 22 (66.7)  4 (12.1)  6 (18.2)  1 (3.0) | 21 (65.6)  -  9 (28.1)  2 (6.3) |
| **Occupational status, n (%)** |  |  |
| Working/student | 29 (87.9) | 30 (93.8) |
| Sick-leave | 1 (3.0) | 2 (6.3) |
| Unemployed | 1 (3.0) | - |
| Parental leave  Other | 1 (3.0)  1 (3.0) | -  - |
|  |  |  |
| **Self-reported financial status, n (%)**  Very bad | 6 (18.2) | 10 (31.3) |
| Bad | 9 (27.3) | 9 (28.1) |
| Neither good or bad | 10 (30.3) | 7 (21.9) |
| Good | 6 (18.2) | 5 (15.6) |
| Very good | 2 (6.1) | 1 (3.1) |
|  |  |  |
| **Duration of gambling problems, n (%)**  >1 year  1-2 years  3-5 years  6-10 years  More than 10 years | -  9 (27.3)  11 (33.3)  10 (30.3)  3 (9.1) | 1 (3.1)  8 (25.0)  8 (25.0)  4 (12.5)  11 (34.4) |
| **Gambling disorder severity, n (%)**  Mild  Moderate  Severe  **Previous treatment for gambling problems, n (%)** | 5 (15.2)  21 (63.6)  7 (21.2) | 8 (22.2)  16 (44.4)  12 (33.3) |
| Yes  No | 11 (33.3)  22 (67.7) | 11 (34.4)  21 (65.6) |
| **Smoker, n (%)**  Yes  No | 5 (15.2)  28 (84.8) | 4 (12.5)  28 (87.5) |
|  |  |  |

*Notes*.

^1^Internet-delivered Cognitive Behavioral Therapy

^2^Internet-delivered Motivational Interviewing

^3^Demographical data (except for Gambling disorder severity) were missing from four of the 36 participants in the Intention to Treat sample as they dropped out before answering the demographics questionnaire.

^4^Statistically significant difference between treatment groups, fishers exact test, *p* < 0.01.
