# Supplementary material for: Internet-delivered therapist-assisted cognitive behavioral therapy for gambling disorder: a randomized controlled trial
Source: Front Psychiatry. 2023 Dec 11;14:1243826. doi: 10.3389/fpsyt.2023.1243826 (PMC10749366; doi:10.3389/fpsyt.2023.1243826)
Supplement: Supplementary file 2 [file Table_2.DOCX]

**Supplemental Table 2.** Observed primary and secondary outcomes (Intention To Treat sample).

| **Measure** | Baseline^1^ | 2 | 3 | 4 | 5 | 6 | 7 | 8 | Post | 6 months |
| --- | --- | --- | --- | --- | --- | --- | --- | --- | --- | --- |
| **NODS**  ICBT^2^      IMI^3^    **Amount bet/week**^4,5^  ICBT      IMI  **Minutes gambled/week**^4^  ICBT      IMI    **PHQ-9**  ICBT      IMI  **GAD-7**  ICBT      IMI  **GBQ**  ICBT      IMI  **BBQ**  ICBT      IMI | 1.9  (2.7)  1.2  (1.9)  221.9  (649.6)  202.1  (821.8)  127.5  (437.6)  116.9  (385.4)  5.5  (3.5)  6.9  (5.3)  5.5  (4.9)  5.8  (5.2)  68.8  (24.0)  67.2  (22.8)  51.4  (20.3)  53.3  (17.0) | 101.9  (494.0)  185.8  (584.9)  183.6  (839.8)  62.0  (191.8)  5.2  (4.8)  5.1  (4.6) | 1.3  (2.2)  0.7  (1.7)  49.4  (204.1)  34.4  (120.8)  26.0  (90.0)  21.0  (68.4)  4.2  (4.3)  4.8  (3.6) | 240.2  (1179.1)  47.6  (223.1)  32.5  (113.7)  28.6  (134.3)  4.4  (4.8)  4.0  (3.9) | 1.0  (1.6)  0.6  (1.3)  8.5  (37.8)  102.0  (464.3)  6.3  (25.0)  36.0  (124.9)  4.3  (4.6)  3.4  (3.3)  3.6  (4.7)  3.1  (3.3)  57.1  (25.5)  54.3  (28.1) | 4.4  (18.7)  43.7  (198.4)  4.8  (18.7)  25.2  (87.3)  3.2  (3.1)  3.0  (2.4) | 0.6  (1.0)  0.7  (1.4)  5.3  (20.4)  50.6  (163.5)  4.3  (14.3)  27.1  84.8  3.2  (3.4)  2.6  (2.4) | 1.5  (6.5)  0.0  (0.0)  1.7  (7.1)  0.0  0.0  2.7  (3.3)  2.3  (2.1) | 0.2  (0.4)  0.3  (1.0)  2.3  (7.5)  200.2  (801.0)  1.4  (6.4)  41.6  (166.3)  3.4  (3.9)  1.3  (1.6)  2.8  (4.1)  1.5  (2.2)  54.3  (24.1)  42.1  (24.9)  63.0  (23.6)  67.2  (26.2) | 1.1  (2.4)  1.0  (2.5)  544.7  (2245.8)  47.1  (118.0)  58.8  (242.5)  42.3  (123.2)  5.0  (7.1)  3.1  (3.8)  4.1  (5.4)  2.9  (4.0)  45.5  (24.0)  36.0  (18.5)  57.6  (29.5)  60.2  (24.6) |
|  |  |  |  |  |  |  |  |  |  |  |

Data are shown as mean (standard deviation).

^1^Treatment start, followed by each week in treatment, post-treatment and 6-month follow-up.

^2^Internet-delivered Cognitive Behavioral Therapy

^3^Internet-delivered Motivational Interviewing

^4^Measured by the Gambling Timeline Follow Back.

^5^Presented in US $. Originally stated in Swedish (SEK; Exchange rate June 1, 2023).
