# Supplementary material for: Internet-delivered therapist-assisted cognitive behavioral therapy for gambling disorder: a randomized controlled trial
Source: Front Psychiatry. 2023 Dec 11;14:1243826. doi: 10.3389/fpsyt.2023.1243826 (PMC10749366; doi:10.3389/fpsyt.2023.1243826)
Supplement: Supplementary file 3 [file Table_3.docx]

**Supplemental Table 3.** Differences post-treatment and at 6-month follow-up between the ICBT^1^ and the IMI^2^ treatment in the Intention To Treat sample.

| **Measure** | **Post-treatment^3^** | **p-value^4^** | **6-month follow-up^5^** | **p-value^6^** |
| --- | --- | --- | --- | --- |
| **NODS**    **Amount bet/week**^7,8^    **Minutes gambled/week**^7^      **PHQ-9**    **GAD-7**  **GBQ**    **BBQ** | -0.1  [-0.4 – 0.2]  211.9  [-143.0 – 566.9]  19.7  [-32.2 – 71.6]  -1.3  [-2.8 – 0.3]  -1.0  [-3.0 – 1.1]  -9.7  [-23.9 – 4.5]  1.2  [-12.2 – 14.5] | 0.512  0.234  0.437  0.104  0.344  0.175  0.861 | -1.1  [-2.8 – 0.5]  -537.3  [-1612.7 – 538.2]  -43.2  [-197.9 – 111.5]  -1.7  [-5.7 – 2.3]  -0.8  [-3.7 – 2.2]  -7.1  [-20.2 – 6.0]  2.1  [-12.7 – 16.9] | 0.169  0.317  0.573  0.388  0.593  0.281  0.778 |
|  |  |  |  |  |

Data are shown as mean (95% confidence interval [CI]).

^1^Internet-delivered Cognitive Behavioral Therapy

^2^Internet-delivered Motivational Interviewing

^3^Model estimated mean difference post-treatment. Positive values indicate a lower value in the ICBT treatment arm.

^4^Calculated using estimated mean differences post-treatment.

^5^Model estimated mean difference at 6-month follow-up. Positive values indicate a lower value in the ICBT treatment arm.

^6^Calculated using estimated mean differences post-treatment.

^7^Measured by the Gambling Timeline Follow Back.

^8^Presented in US $. Originally stated in Swedish (SEK; Exchange rate June 1, 2023).
