# Supplementary material for: Internet-delivered therapist-assisted cognitive behavioral therapy for gambling disorder: a randomized controlled trial
Source: Front Psychiatry. 2023 Dec 11;14:1243826. doi: 10.3389/fpsyt.2023.1243826 (PMC10749366; doi:10.3389/fpsyt.2023.1243826)
Supplement: Supplementary file 4 [file Table_4.DOCX]

**Supplemental Table 4.** Observed values at first visit, baseline, post-treatment and 6-month follow-up for the total Intention To Treat sample.

| Measure | First visit | Baseline | Post | 6-months |
| --- | --- | --- | --- | --- |
| **NODS**    **Amount bet/week**^1,2^    **Minutes gambled/week**^1^      **PHQ-9**    **GAD-7**  **GBQ**    **BBQ** | 4.8 (3.1)  -  -  9.6 (6.0)  7.6 (5.5)  72.5 (19.3)  42.0 (21.2) | 1.6 (2.4)  211.8 (735.7)  122.1 (408.2)  6.2 (4.5)  5.6 (5.0)  68.0 (23.3)  52.3 (18.7) | 0.2 (0.7)  85.6 (519.5)  18.3 (107.9)  2.5 (3.3)  2.2 (3.5)  49.0 (24.9)  64.9 (24.5) | 1.1 (2.4)  275.7 (1520.6)  49.9 (185.0)  4.0 (5.6)  3.4 (4.7)  40.4 (21.4)  59.0 (26.6) |
|  |  |  |  |  |

Data are shown as mean (standard deviation).

^1^Measured by the Gambling Timeline Follow Back.

^2^Presented in US $. Originally stated in Swedish (SEK; Exchange rate June 1, 2023)
