# Supplementary material for: Internet-delivered therapist-assisted cognitive behavioral therapy for gambling disorder: a randomized controlled trial
Source: Front Psychiatry. 2023 Dec 11;14:1243826. doi: 10.3389/fpsyt.2023.1243826 (PMC10749366; doi:10.3389/fpsyt.2023.1243826)
Supplement: Supplementary file 5 [file Table_5.docx]

**Supplemental Table 5.** Estimated mean effects of time between baseline to post-treatment and post-treatment to 6-month follow-up in the total Intention To Treat sample.

| **Measure** | **Baseline – Post^1^** | **p-value^2^** | **Effect size^3^** | **Post - 6-months^4^** | **p-value^5^** | **Effect size^6^** |
| --- | --- | --- | --- | --- | --- | --- |
| **NODS**    **Amount bet/week**^7,8^    **Minutes gambled/week**^7^    **PHQ-9**    **GAD-7**  **GBQ**    **BBQ^9^** | 1.2  [0.6 – 1.8]  140.8  [-118.5 – 400.2]  99.0  [-12.8 – 210.9]  3.8  [2.2 – 5.3]  3.4  [2.2 – 4.7]  20.1  [14.4 – 25.8]  -11.1  [-16.0 – -6.1] | < 0.001  0.284  0.082  < 0.001  < 0.001  < 0.001  < 0.001 | 0.50  [-1.16 – 2.00]  N/A  N/A  0.84  [-1.08 – 2.58]  0.69  [-1.20 – 2.38]  0.8623  [-0.72 – 2.33]  0.59  [-0.62 – 1.69] | -1.0  [-1.8 – -0.2]  -179.7  [-699.6 – 340.3]  -29.4  [-100.5 – 41.6]  -2.6  [-4.2 – -1.1]  -1.6  [-2.9 – -0.3]  7.1  [-2.2 – 16.5]  5.2  [-0.9 – 11.4] | 0.016  0.488  0.405  0.001  0.018  0.131  0.092 | 0.42  [-1.75 – 2.43]  N/A  N/A  0.59  [-0.82 – 1.85]  0.32  [-0.98 – 1.51]  N/A  N/A |
|  |  |  |  |  |  |  |

Data are shown as mean (95% confidence interval [CI]).

^1^Model estimated mean difference with 95% confidence intervals between baseline and post-treatment for the total sample. Positive values indicate a reduction from baseline.

^2^Calculated for baseline – post effect.

^3^Effect size with 95% confidence intervals calculated between baseline and post-treatment for significant effects.

^4^Model estimated mean difference with 95% confidence intervals between post-treatment and 6-month follow-up for the total sample. Positive values indicate a reduction from baseline.

^5^Calculated for post – 6-months effect.

^6^Effect size with 95% confidence intervals calculated between post-treatment and 6-month follow-up for significant effects.

^7^Measured by the Gambling Timeline Follow Back.

^8^Presented in US $. Originally stated in Swedish (SEK; Exchange rate June 1, 2023).

^9^Higher scores indicate better Quality of Life on the BBQ.
